# Study on influencing factors of age-adjusted Charlson comorbidity index in patients with Alzheimer's disease based on machine learning model

**DOI:** 10.3389/fmed.2025.1497662

**Published:** 2025-01-27

**Authors:** Jian Ding, Zheng Long, Yiming Liu, Min Wang

**Affiliations:** ^1^Department of Neurology, Shandong Public Health Clinical Center, Shandong University, Jinan, China; ^2^Department of Neurology, Qilu Hospital, Shandong University, Jinan, China; ^3^Department of Medical Affairs, Xuanwu Hospital, Capital Medical University, Beijing, China; ^4^Department of Neurology, The Second Hospital of Shandong University, Jinan, Shandong, China

**Keywords:** Alzheimer's disease, Charlson Comorbidity Index (CCI), machine learning, dementia disease, MIMIC-IV database

## Abstract

**Background:**

Alzheimer's disease (AD) is a widespread neurodegenerative disease, often accompanied by multiple comorbidities, significantly increasing the risk of death for patients. The age adjusted Charlson Comorbidity Index (aCCI) is an important clinical tool for measuring the burden of comorbidities in patients, closely related to mortality and prognosis. This study aims to use the MIMIC-V database and various regression and machine learning models to screen and validate features closely related to aCCI, providing a theoretical basis for personalized management of AD patients.

**Methods:**

The research data is sourced from the MIMIC-V database, which contains detailed clinical information of AD patients. Multiple logistic regression, LASSO regression, random forest, Support Vector Machine (SVM), and Extreme Gradient Boosting (XGBoost) models were used to screen for feature factors significantly correlated with aCCI. By comparing model performance, evaluating the classification ability and prediction accuracy of each method, and ultimately selecting the best model to construct a regression model and a nomogram. The model performance is evaluated through classification accuracy, net benefit, and robustness. The feature selection results were validated by regression analysis.

**Results:**

Multiple models have performed well in classifying aCCI patients, among which the model constructed using LASSO regression screening feature factors has the best performance, with the highest classification accuracy and net benefit. LASSO regression identified the following 11 features closely related to aCCI: age, respiratory rate, base excess, glucose, red blood cell distribution width (RDW), alkaline phosphatase (ALP), whole blood potassium, hematocrit (HCT), phosphate, creatinine, and mean corpuscular hemoglobin (MCH). The column chart constructed based on these feature factors enables intuitive prediction of patients with high aCCI probability, providing a convenient clinical tool.

**Conclusion:**

The results of this study indicate that the features screened by LASSO regression have the best predictive performance and can significantly improve the predictive ability of aCCI related comorbidities in AD patients. The column chart constructed based on this feature factor provides theoretical guidance for personalized management and precise treatment of AD patients.

## 1 Introduction

Alzheimer's disease (AD) is a neurodegenerative disorder characterized by the progressive and irreversible decline in cognitive abilities and stands as the primary cause of dementia (1–3). The World Health Organization estimated that over 55 million people worldwide were living with dementia, and this number was expected to double every 20 years, reaching its peak around 2050 (4, 5). As the global population ages, AD is anticipated to pose significant challenges to patients, families, healthcare systems, and societies worldwide, emerging as one of the most serious and formidable public health threats of the 21st century (1). Moreover, as age advances, AD patients become more prone to a range of comorbidities, including cardiovascular and cerebrovascular diseases, diabetes, and infections (6–8). Studies had shown a link between the presence of these comorbidities and mortality in AD patients, with those having more comorbidities experiencing higher mortality rates (9, 10).

The Charlson Comorbidity Index (CCI) is a standardized score developed by Mary E. Charlson to measure the degree of comorbidity, calculated through a simple weighted sum of comorbidity item scores, and is widely regarded as the gold standard for predicting patient prognosis in clinical research (11). Building on the original CCI, researchers developed the age-adjusted Charlson Comorbidity Index (aCCI), which has been shown to closely correlate with mortality and serves as an effective predictor of clinical outcomes across various diseases (12). Our study employed the more comprehensive aCCI to measure the degree of comorbidity.

With the widespread adoption of electronic health record systems, the emergence of large medical datasets like the MIMIC-IV database provides rich data resources and unprecedented research opportunities for retrospective studies, enabling researchers to conduct deeper analyses of patients' clinical features, disease progression, and treatment efficacy (13). In the face of complex and large datasets, machine learning is widely used in retrospective studies for its exceptional ability to identify complex and non-linear relationships among numerous prognostic variables (14).

Previous studies reported higher mortality in AD patients with high CCI (1, 9). However, there is still a lack of research on the factors influencing high aCCI in AD patients admitted to the ICU. Therefore, using aCCI as the research outcome in AD patients and identifying the factors that contribute to a high aCCI holds significant research value. Similar to previous studies, our research included patient demographic information, vital signs, and rating scales (1, 9, 15). To explore additional factors, we chose to include all laboratory test results in our analysis, rather than selectively choosing specific tests. Confronted with numerous factors, we employed four types of machine learning: LASSO regression, Random Forest, Support Vector Machine (SVM), and eXtreme Gradient Boosting (XGBoost) to identify specific disease factors. Simultaneously, considering the verification of the machine learning model, we also used a multivariate logistic regression model. To fairly assess the predictive ability of the characteristic factors identified by each model, we incorporated them into a regression model for validation. Additionally, the final selected regression model was used to construct a nomogram to predict the probability of high aCCI in AD patients. Our study aimed to identify the factors that contribute to severe comorbidities in AD patients and to assist clinicians in developing personalized treatments to enhance their quality of life.

## 2 Materials and methods

### 2.1 Data sources and study patients

**Figure 1:**
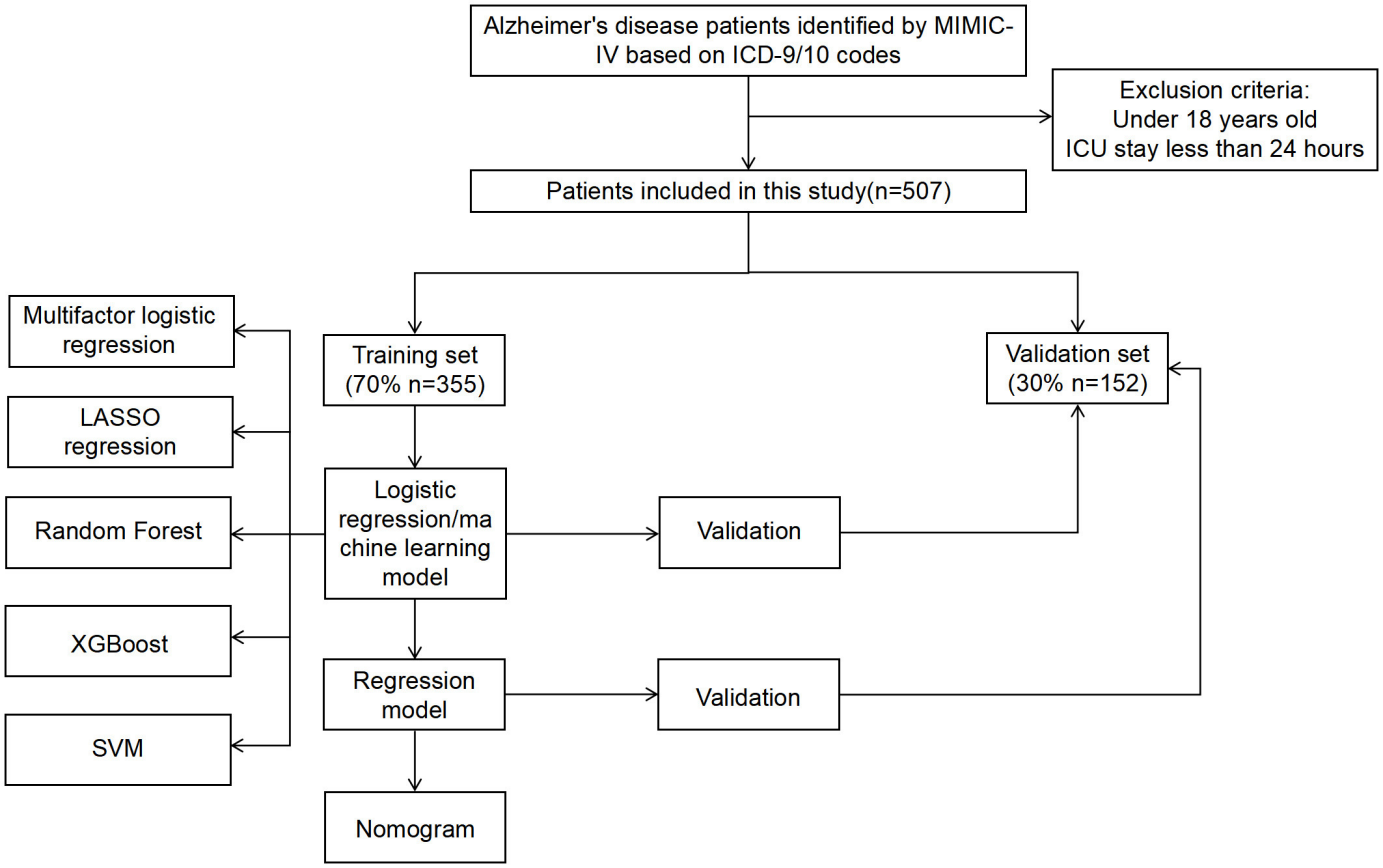
The workflow of our study.

Our study conducted a retrospective analysis using the MIMIC-IV database. MIMIC-IV is a publicly accessible database, meticulously curated under the supervision of the Institutional Review Boards of the Massachusetts Institute of Technology (MIT) and Beth Israel Deaconess Medical Center, containing comprehensive high-quality clinical data spanning from 2008 to 2019. Diagnosis headings containing “Alzheimer's disease” were selected from MIMIC-IV, which included patients with ICD versions 9 and 10. Patients younger than 18 years old or those with an ICU stay of < 24 h were excluded from the study. In our study, 507 patients diagnosed with Alzheimer's disease(AD) who met the inclusion criteria were gathered. The work flow of our study is shown in Figure 1.

### 2.2 Patient data extraction

Based on the AD patient's ID (subject_id), the corresponding demographics, vital signs, laboratory test indicators, and scoring scales were extracted from the MIMIC-IV database. Data corresponding to AD patients were extracted from the MIMIC-IV database using the hospitalization ID (subject_id). The extracted data included demographics (age, gender), vital signs [temperature, heart rate (Heartrate), respiratory rate(Resprate), Systolic Blood Pressure (SBP), Diastolic Blood Pressure (DBP)], laboratory test results, rating scales, and the age-adjusted Charlson Comorbidity Index (aCCI). Laboratory tests encompassed all relevant indicators associated with AD patients. For scoring scales, scores recorded on the first day of admission were analyzed, with a focus on the Sequential Organ Failure Assessment (SOFA), Systemic Inflammatory Response Syndrome (SIRS), Logistic Organ Dysfunction Scoring System (LODS), and Glasgow Coma Scale (GCS).

### 2.3 Patient data processing

The MIMIC-IV database often presents challenges related to missing data, which, if not appropriately handled, can lead to significant bias in the analysis. To address this issue, variables with missing data exceeding 40% were excluded from the analysis. For variables with < 5% missing data, imputation was performed based on the nature of their distribution: for continuous variables with a normal distribution, missing values were replaced by the mean of the patient cohort, while for those with a skewed distribution, the median was used for imputation (16). For variables with more than 5% but < 40% missing data, the “mice” package in R was employed for multiple imputation. This method addresses the uncertainty of missing values by imputing several plausible values for each missing entry, enhancing the robustness and reliability of subsequent analyses (17–20). To evaluate the comorbidity burden, we used the age-adjusted Charlson Comorbidity Index (aCCI). Patients were categorized into two groups: low comorbidity (aCCI ≤ 5) and high comorbidity (aCCI > 5), based on previously validated thresholds (12, 21). However, in addition to this categorical classification, aCCI was also treated as a continuous variable to provide a more comprehensive evaluation of its influencing factors. This dual approach enabled both grouped comparisons and continuous variable analysis to capture the linear relationships between comorbidities and clinical features. A baseline table was generated using the R “compareGroups” package to assess the distribution of patient variables across the aCCI-low and aCCI-high groups. Variables showing statistically significant differences (*P* < 0.05) between the two groups were selected for further analysis. To explore the factors influencing aCCI, these variables were incorporated into multivariate regression models, where aCCI was analyzed both as a categorical and a continuous variable, providing deeper insights into its associations with clinical features.

### 2.4 Feature variable filtering and nomogram construction

The R “tidyverse” package was utilized to randomly split the patients into training and validation sets at a 7:3 ratio. The training set was employed to develop the machine learning model, while the validation set was used to assess its performance. To identify the most critical feature variables for predicting aCCI groups, our study employed a multivariate logistic regression model alongside three machine learning models: LASSO regression, Random Forest, SVM, and XGBoost.

Before constructing the models, Variance Inflation Factor (VIF) analysis was conducted to assess multicollinearity among the selected variables (Supplementary Table 1). All variables demonstrated VIF values below 10, confirming low multicollinearity and ensuring the statistical independence of the predictors. This step reduced potential bias and enhanced the stability of subsequent model training.

The discriminative ability of each model was evaluated by calculating the area under the receiver operating characteristic (ROC) curve (AUC). Among the models, LASSO regression achieved competitive performance, leveraging its regularization technique to address potential overfitting and optimize feature selection. The optimal feature variables identified from the model with the highest AUC were then incorporated into separate regression models for further evaluation.

The accuracy of these models was further assessed using ROC curves and calibration curves to ensure consistency between predicted and observed outcomes. Additionally, decision curve analysis (DCA) was performed to evaluate the clinical net benefit of each model, offering insights into their practical applicability in clinical settings (22).

The variables derived from the optimal regression model were identified as key factors influencing the occurrence of severe comorbidities in AD patients. A predictive nomogram was constructed using these variables, providing a visual and interpretable tool for estimating the likelihood of patients developing severe comorbidities. This nomogram has the potential to assist clinicians in early identification of high-risk patients and facilitate personalized management strategies, ultimately improving patient outcomes.

### 2.5 Statistical analysis

Statistical analysis was conducted using R version 4.3.2. Categorical variables were expressed as counts and percentages, with group comparisons performed using the chi-square test (χ^2^) or Fisher's exact test. Continuous variables were presented as mean ± standard deviation (SD) for normally distributed data and compared using one-way analysis of variance (ANOVA). For non-normally distributed data, variables were expressed as median (interquartile range, IQR) and analyzed using the Wilcoxon rank-sum test. A *P*-value of < 0.05 was considered statistically significant.

Sensitivity analyses were conducted to assess the robustness of the results, including varying the aCCI thresholds. Interaction effects between variables were explored using regression models to identify any modifying factors. Benjamini-Hochberg correction was applied to control the false discovery rate (FDR) for multiple comparisons, with an adjusted *P*-value of < 0.05 considered significant.

## 3 Results

### 3.1 Study data characteristics

As illustrated in Supplementary Figure 1, our study included 507 patients with Alzheimer's disease (AD) extracted from the MIMIC-IV database. Each patient had 509 factors collected, and the proportion of missing data for each factor was analyzed (Supplementary Table 2). After applying the inclusion criteria, 52 factors were retained for further analysis. Consistent with prior studies, an aCCI value of 5 was selected as the threshold for grouping, categorizing 398 patients into the aCCI-high group and 109 patients into the aCCI-low group. The distribution of the age-adjusted Charlson Comorbidity Index (aCCI) is shown in Supplementary Figure 2. The histogram demonstrates a right-skewed distribution, with most patients concentrated between scores of 5 and 10. The distribution of key feature variables across different aCCI levels is illustrated in Supplementary Figure 3. The boxplots highlight the varying patterns of feature variables, such as Glucose, RDW, and Phosphate, as aCCI increases. Baseline characteristics of the patients stratified by aCCI grouping were generated using the “compareGroups” package in R (Supplementary Table S2). As shown in Table 1, AD patients in the aCCI-high group exhibited significantly higher levels of Age, Resprate, Anion Gap, Glucose, RDW, Alkaline Phosphatase, Potassium (Whole Blood), Urea Nitrogen, Phosphate, and Creatinine (*P* < 0.05 for all), while their MCHC, Base Excess, Hematocrit, Hemoglobin, and MCH were significantly lower (*P* < 0.05 for all, adjusted using Benjamini-Hochberg correction). These significant differences between the two groups demonstrate that the aCCI-based grouping is representative of clinically meaningful differences in the patient population. The 15 significant differentiating factors, apart from aCCI itself, were selected for inclusion in subsequent analyses.

**Table 1:** Baseline table of differentiating factors between aCCI groups in AD patients.

	**All**	**aCCI-low**	**aCCI-high**	***P*-overall**	**Benjamini-Hochberg *p***
	***n*** = **507**	***n*** = **109**	***n*** = **398**		
Age	85.00 (79.00; 89.50)	81.00 (74.00; 89.00)	85.00 (80.00; 90.00)	0.009	0.009
Resprate	18.00 (16.00; 21.00)	18.00 (16.00; 19.00)	18.00 (16.00; 21.00)	0.001	0.003
aCCI	7.00 (6.00; 8.00)	5.00 (5.00; 5.00)	7.00 (6.00; 9.00)	< 0.001	< 0.001
MCHC	32.90 (31.80; 33.70)	33.00 (32.00; 34.30)	32.80 (31.70; 33.60)	0.032	0.034
Base excess	0.00 (−2.00; 1.00)	0.00 (−1.00; 2.00)	0.00 (−3.00; 1.00)	0.022	0.031
Anion gap	15.00 (13.00; 17.00)	14.00 (13.00; 16.00)	15.00 (13.00; 18.00)	0.016	0.026
Glucose	125.00 (102.00; 164.50)	113.00 (93.00; 134.00)	129.00 (103.25; 181.00)	< 0.001	< 0.001
RDW	14.10 (13.30; 15.10)	13.90 (13.20; 14.60)	14.20 (13.40; 15.17)	0.003	0.007
Alkaline phosphatase	82.00 (64.00; 111.00)	75.00 (60.00; 96.00)	84.00 (65.00; 111.00)	0.011	0.015
Potassium (whole blood)	4.20 (3.70; 4.70)	4.10 (3.60; 4.50)	4.20 (3.80; 4.80)	0.019	0.023
Hematocrit	37.20 (33.50; 40.60)	38.90 (34.60; 41.10)	36.95 (33.32; 40.40)	0.018	0.021
Urea nitrogen	23.00 (17.00; 31.50)	19.00 (16.00; 25.00)	23.00 (17.00; 34.00)	< 0.001	< 0.001
Hemoglobin	12.20 (11.00; 13.40)	12.70 (11.60; 13.70)	12.00 (10.70; 13.30)	0.003	0.006
Phosphate	3.40 (2.80; 3.90)	3.10 (2.60; 3.70)	3.40 (2.90; 4.00)	< 0.001	< 0.001
Creatinine	1.00 (0.80; 1.40)	0.90 (0.80; 1.10)	1.10 (0.80; 1.40)	< 0.001	< 0.001
MCH	30.20 (28.80; 31.50)	30.60 (29.50; 31.80)	30.10 (28.60; 31.50)	0.015	0.022

### 3.2 Feature variable

**Figure 2:**
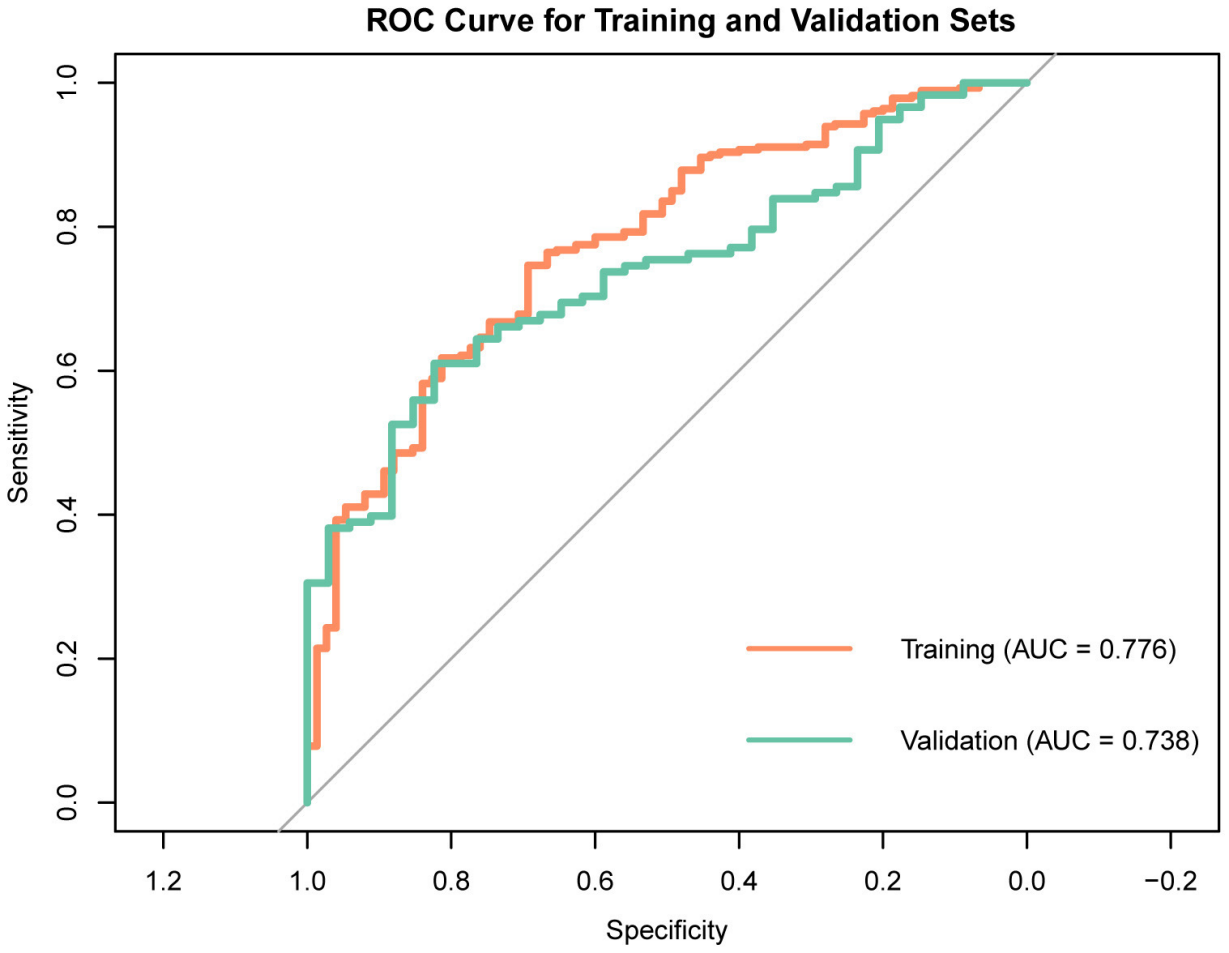
ROC curve of multivariate logistic regression model.

To maintain the study's objectivity, all patients were randomly allocated into a training set (70%, *n* = 355) and a validation set (30%, *n* = 152). The training set was utilized to build models using Multivariate logistic regression, LASSO regression, Random Forest, XGBoost, and SVM. The Multivariate logistic regression model identified three variables with *P* values < 0.05 among the 15 variables: Age, Glucose, and Phosphate (Table 2). The ROC curve of the Multivariate logistic regression model indicates that the AUC in the training set is 0.776, while in the validation set, it is 0.738 (Figure 2).

**Table 2:** Summary of multivariate logistic regression.

	**Estimate**	**Std. error**	***Z*-value**	***P*-value**
Age	0.05	0.02	3.076	0.002
Resprate	0.07	0.04	1.816	0.069
MCHC	0.01	0.28	0.053	0.958
Base excess	−0.03	0.04	−0.865	0.387
Anion gap	−0.03	0.05	−0.594	0.552
Glucose	0.01	0.00	2.612	0.009
RDW	0.04	0.11	0.381	0.703
Alkaline phosphatase	0.01	0.00	1.609	0.108
Potassium (whole blood)	0.16	0.20	0.769	0.442
Hematocrit	−0.07	0.23	−0.280	0.780
Urea nitrogen	−0.01	0.01	−1.003	0.316
Phosphate	0.57	0.19	2.995	0.003
Creatinine	0.83	0.51	1.635	0.102
Hemoglobin	0.09	0.71	0.132	0.895
MCH	−0.13	0.07	−1.775	0.076

**Figure 3:**
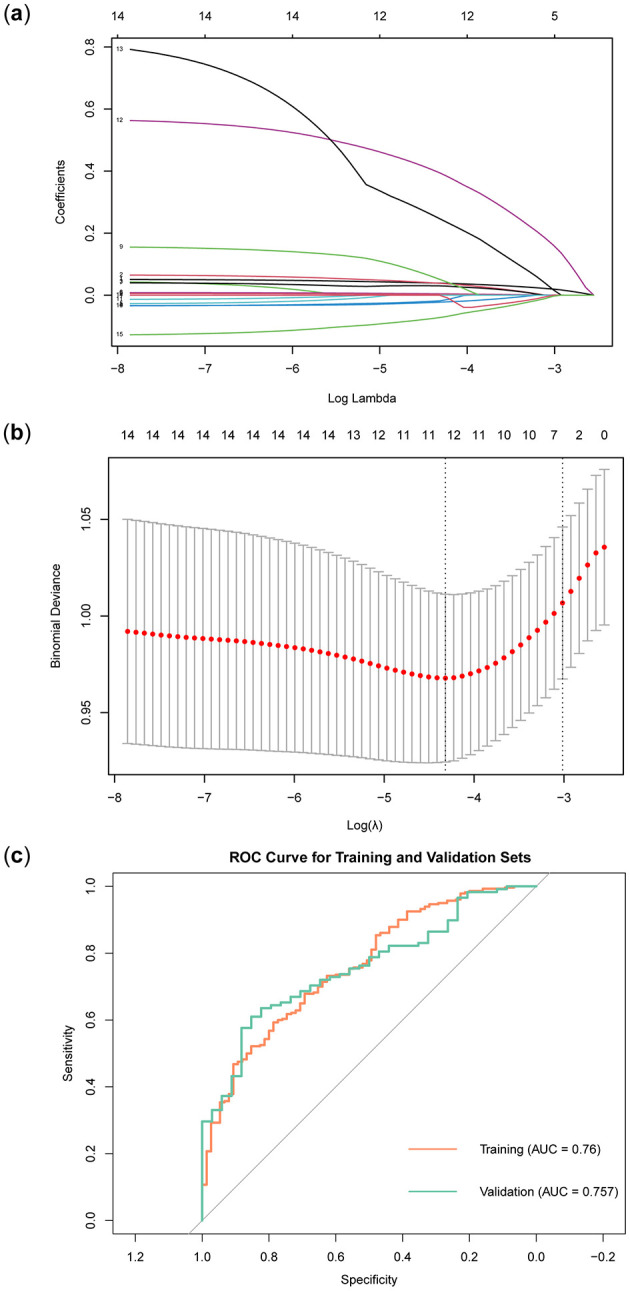
Construction and validation of LASSO regression model. **(A)** LASSO regression path diagram. **(B)** LASSO regression model cross-validation curve. **(C)** LASSO regression model ROC curve.

In the training set, the LASSO regression model tracks the path of coefficient changes with the regularization parameter λ (Figure 3A), and the optimal λ value is determined through 10-fold cross-validation (Figure 3B). The two vertical dashed lines represent the λ value corresponding to the minimum deviation within one standard error and the λ value that yields the simplest model with the fewest features. We selected the λ value corresponding to the minimum deviation (λ = 0.0133127), resulting in 11 feature variables (Table 3). The ROC curve of the LASSO regression model revealed that the AUC in the training set was 0.76, while in the validation set, it was 0.757 (Figure 3C).

**Table 3:** 11 feature variables and their coefficients identified by the LASSO regression mode.

**Feature**	**Coeff**
Age	0.038958091
Resprate	0.038492811
Base excess	−0.022410513
Glucose	0.004535585
RDW	0.03019722
Alkaline phosphatase	0.002674139
Potassium (whole blood)	0.05159612
Hematocrit	−0.018235899
Phosphate	0.394963953
Creatinine	0.247257609

**Figure 4:**
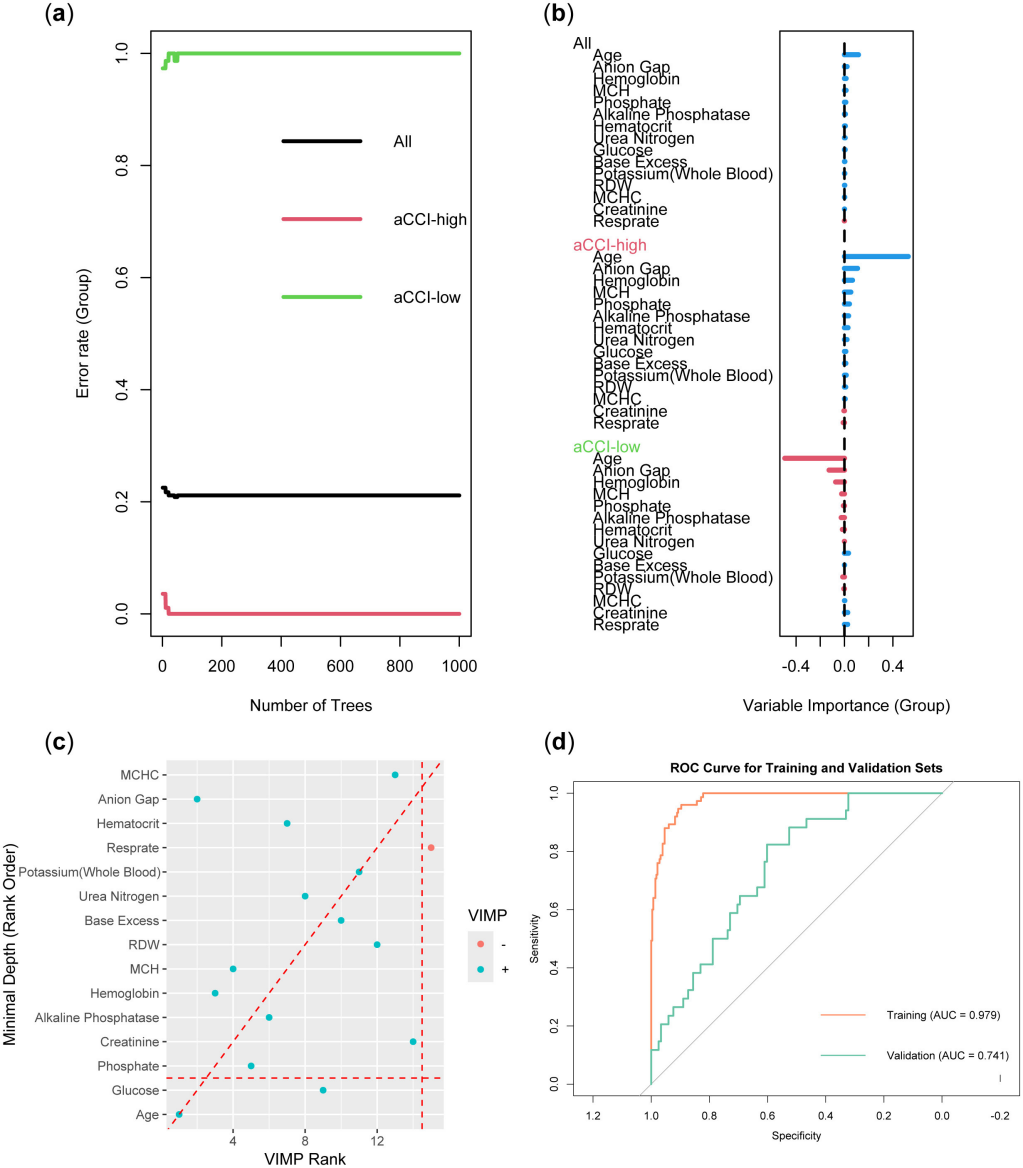
Construction and validation of Random Forest models. **(A)** Error rate as a function of the number of trees. **(B)** Variable importance plot. **(C)** Minimum depth vs. variable importance (VIMP) plot. **(D)** ROC curve of the model.

The Random Forest model was constructed using the training set data. To prevent overfitting, we set the number of trees to 100, the node size to 15, and employed sampling with replacement. Figure 4A demonstrated that as the number of trees increased, the error rate gradually decreased and stabilized. This indicated that the random forest model could achieve stable classification with a sufficient number of trees. Notably, the aCCI-high group exhibited the lowest error rate, suggesting that the model performed best in this group and was effective in identifying factors influencing the aCCI-high group. The importance of different variables in the Random Forest model was ranked according to their contribution to model prediction (Figure 4B). The colors distinguished the importance of variables between the aCCI-high and aCCI-low groups. We explored the relationship between minimum depth and variable importance (VIMP) to identify the variables that the model considered could be excluded. The results showed that Resprate was not considered important by the model (Figure 4C). In Figure 4D, the ROC curve of the Random Forest model showed that the AUC value for the training set was 0.979, while the AUC value for the validation set was 0.761.

**Figure 5:**
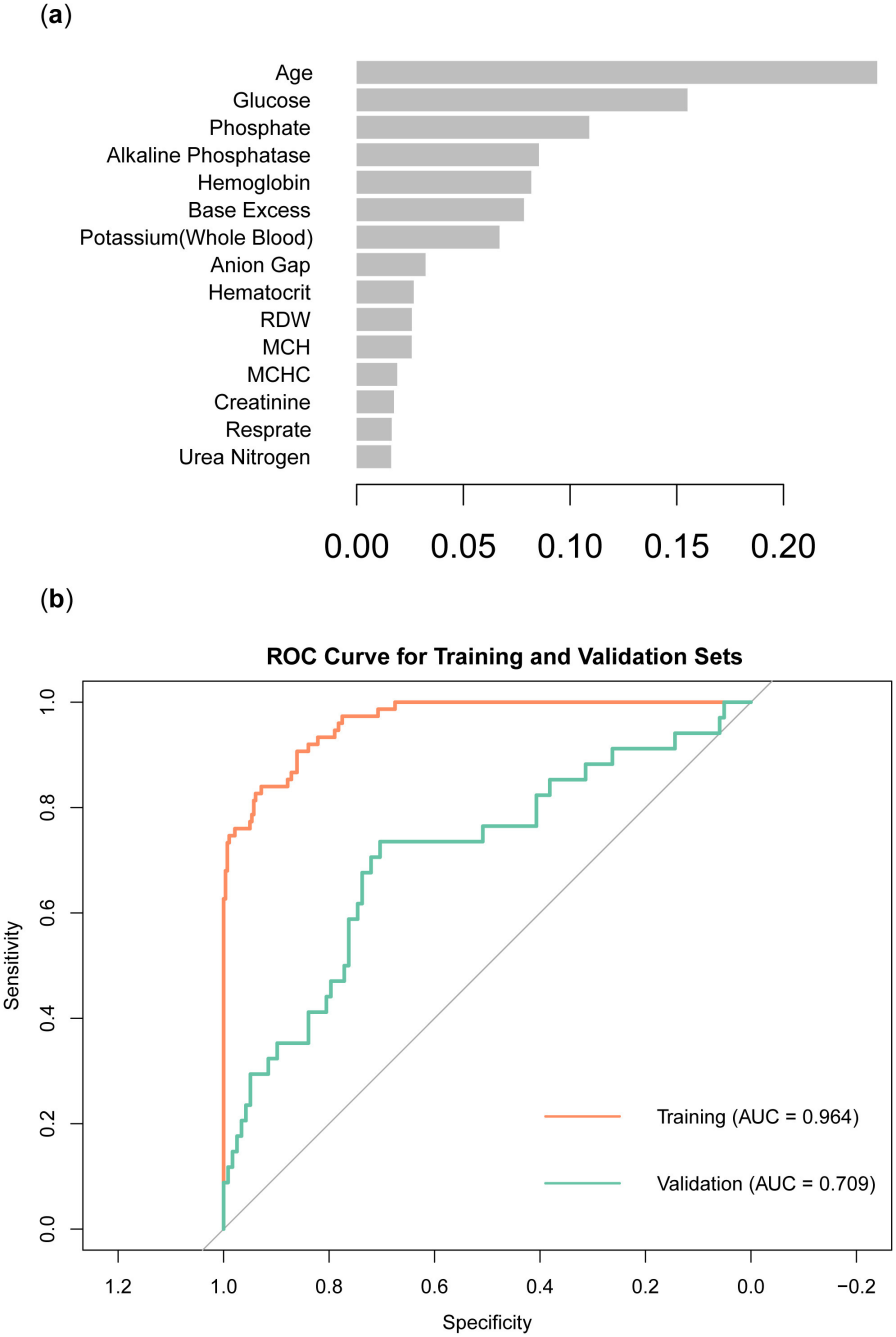
Construction and validation of XGBoost models. **(A)** Ranking of variable importance in the XGBoost model. **(B)** ROC curve of XGBoost model.

In order to obtain a more accurate evaluation of variable importance by the XGBoost model, we used 10-fold cross-validation on the training set to build the model. The model used binary logistic regression as the objective function and applied L1 and L2 regularization (alpha = 1, lambda = 10) to enhance the model's generalization ability. To control model complexity and prevent overfitting, we set the number of iterations to 100, the learning rate to 0.05, and the maximum tree depth to 4. We then calculated the model's scores for the importance of each variable, ranked them, and visualized the results (Figure 5A). The results showed that Age was the most important variable, with the highest score and the greatest contribution to the model's prediction outcomes. Glucose and Phosphate also showed high importance, closely following the leading variable. Other variables of high importance included Alkaline Phosphatase, Hemoglobin, and Base Excess. In contrast, Urea Nitrogen, Respirate, and Creatinine were less important and had a smaller impact on the model. The ROC curve showed that the model had an AUC of 0.964 on the training set and an AUC of 0.709 on the validation set (Figure 5B).

**Figure 6:**
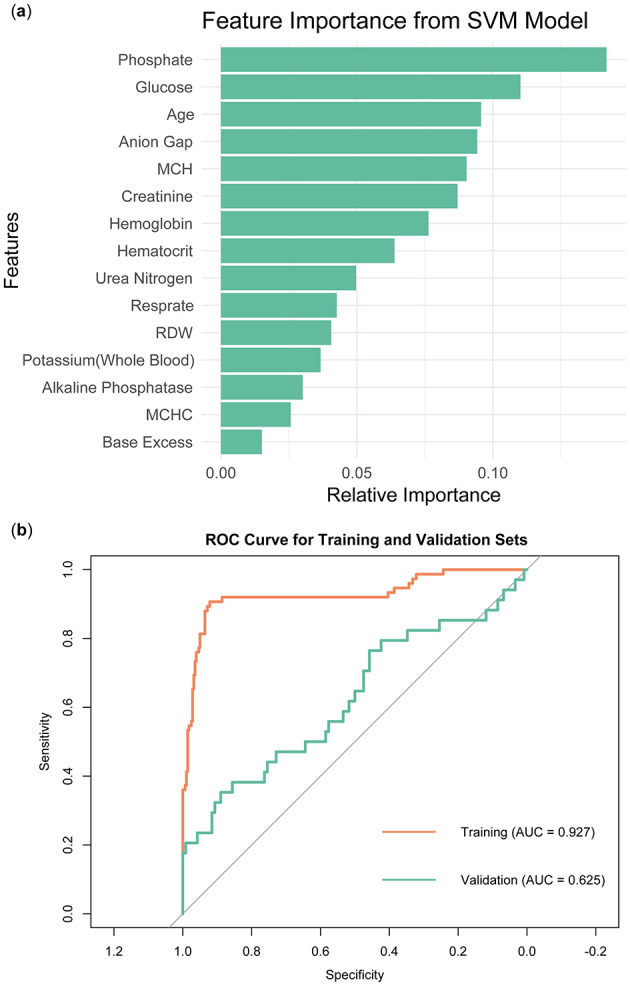
Construction and validation of SVM models. **(A)** Ranking of variable importance in the SVM model. **(B)** ROC curve of SVM model.

In order to identify feature variables through the SVM model, a model was constructed using the radial basis function (RBF) kernel. Similar to LASSO regression, the model's training data set employed cross-validation. Figure 6A illustrated the importance ranking of each variable in the SVM model. Phosphate emerged as the most important variable, with the highest score, indicating its significant contribution to the model's predictive performance. Other features variables included Glucose, Age, and Anion Gap, which also demonstrated high relative importance within the model. Base Excess and MCHC were ranked lower in importance, suggesting they had a smaller impact on the model's predictions. Figure 6B showed that the SVM model performed well on the training set (AUC = 0.927), but its performance on the validation set was significantly lower (AUC = 0.625).

Given that the feature variables identified by each model differ, we needed to compare the AUC of the five models on both the training and validation sets to select the model with better performance for determining the feature variables. From the above results, it was observed that the Multivariate logistic regression model and the LASSO regression model demonstrated stable and strong performance across both the training and validation sets. The Random Forest model performed exceptionally well on the training set, and while its performance on the validation set did not match that of the training set, the AUC remained comparable to the previous two models. However, the performance gap between XGBoost and SVM across the two data sets was more pronounced, with the SVM model showing particularly noticeable discrepancies. Therefore, we selected the results identified by the three models of Multivariate logistic regression, LASSO regression, and Random Forest for further analysis (Table 4).

**Table 4:** Feature variables included in each model for further analysis.

**Multivariate logistic regression**	**LASSO regression**	**Random Forest**
Age	Age	Age
Glucose	Resprate	Anion gap
Phosphate	Base excess	Hemoglobin
	Glucose	MCH
	RDW	Phosphate
	Alkaline phosphatase	Alkaline phosphatase
	Potassium (whole blood)	Hematocrit
	Hematocrit	Urea nitrogen
	Phosphate	Glucose
	Creatinine	Base excess
		Potassium (whole blood)
		RDW
		MCHC
		Creatinine

### 3.3 Regression model

**Figure 7:**
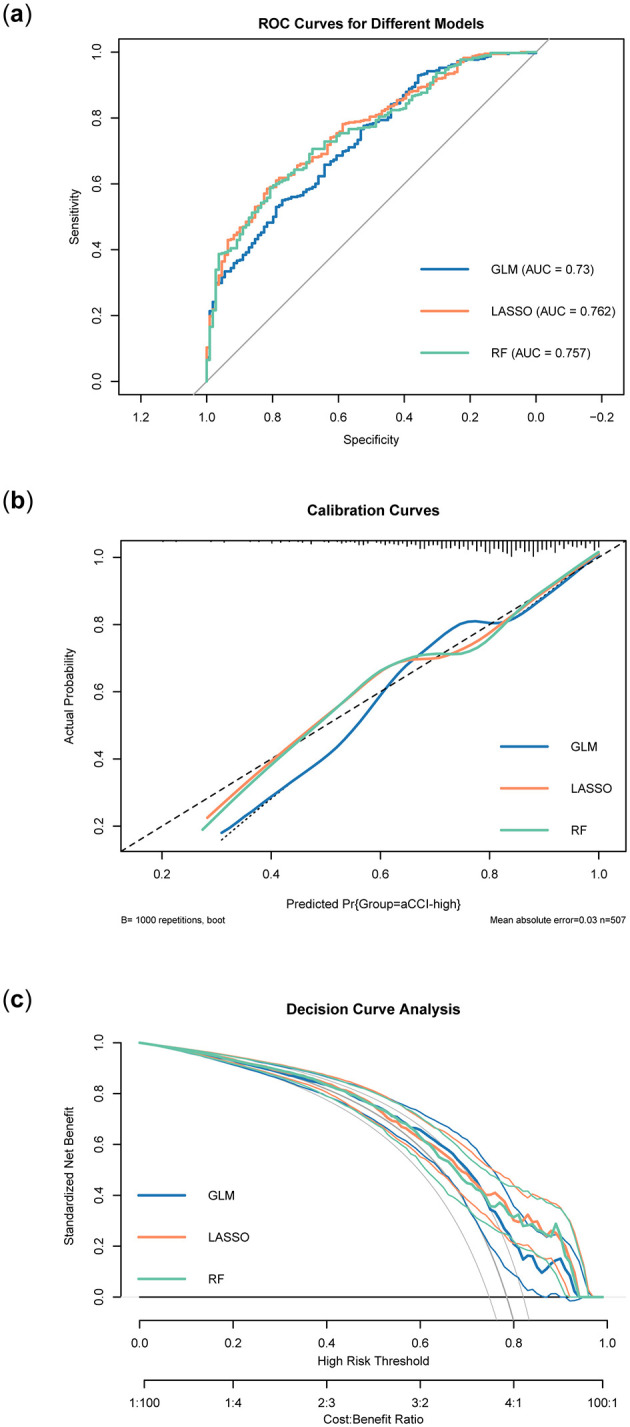
Validation of three regression models. **(A)** ROC curves of different models. **(B)** Calibration curves of different models. **(C)** Decision curves of different models.

Regression models were constructed using the feature variables of the preferred models listed in Table 4. Feature variables included in each model for further analysis. Feature variables included in each model for further analysis and were subsequently named GLM, LASSO, and RF, respectively. Following this, we conducted a comparison of the three regression models using ROC curves, calibration curves, and DCA, evaluating their performance from various dimensions. Figure 7A displayed the ROC curves for the three regression models: GLM, LASSO, and RF. Overall, the LASSO model performed the best among the three, followed by the RF model, while the GLM model's performance was relatively poor. Figure 7B presented the calibration curves for the GLM, LASSO, and RF models, assessing how well the predicted probabilities matched the actual outcomes. The calibration curve for the GLM model showed a deviation from the diagonal, indicating a discrepancy between its predicted probabilities and the actual probabilities. In contrast, the LASSO model's calibration curve was much closer to the diagonal, reflecting more accurate predictions. Similarly, the RF model's calibration curve was also near the diagonal, indicating good calibration performance. Taken together, the LASSO and RF models demonstrated better calibration accuracy than the GLM model, with predicted probabilities aligning more closely with actual outcomes. Figure 7C presented the DCA results for the three models. Comparing their decision curves allowed us to evaluate the clinical net benefits across different risk thresholds. At most high-risk thresholds, the LASSO and RF models demonstrated higher net benefits, indicating greater potential for clinical application. The GLM model performed reasonably well at some lower thresholds. However, its overall net benefit was lower compared to the LASSO and RF models. Overall, the LASSO and RF models provided higher normalized net benefits across various threshold ranges, suggesting superior utility in clinical decision-making. Combining the results from the three evaluations, the LASSO excelled in both AUC and calibration performance, and demonstrated a high clinical net benefit in the decision curve analysis. In contrast, the GLM model's performance was relatively weaker across all three dimensions, particularly in terms of AUC and calibration curve, where its predictive ability and accuracy were inferior to those of the other two models.

### 3.4 Nomogram

Since LASSO demonstrated the best overall performance, we selected the characteristic variables identified by the LASSO regression model to develop a high comorbidity prediction algorithm, as outlined below:


$$\begin{array}{r} \text{Probability of High aCCI}=-4.66284947119117\times \text{Intercept}\\ +\;0.0489336652551905\times \text{Age}+0.0721263172679331\times \text{Resprate}\\ -0.0119413850308944\times \text{Base Excess}\\ +0.00852224585145184\times \text{Glucose}+0.0351643998822672\\ \times \text{RDW}+0.00608867108322709\times \text{Alkaline Phosphatase}\\ +0.0266239100293162\times \text{Potassium}\left(\text{Whole Blood}\right)\\ -0.0318367220265998\times \text{Hematocrit}+0.507495078536365\\ \times \text{Phosphate}+0.520868238172282\\ \times \text{Creatinine}-0.0920164189252346\times \text{MCH} \end{array}$$

**Figure 8:**
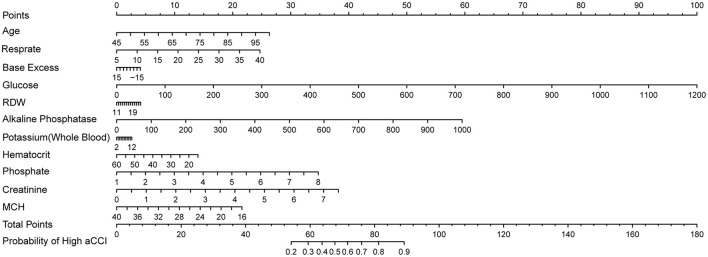
Nomogram based on LASSO.

By visualizing the above model formula, a nomogram could be obtained. This nomogram was constructed based on the LASSO model (Figure 8). Given the strong overall performance of LASSO, we could infer that our nomogram offers reliable performance, aiding clinicians in making more accurate risk assessments and treatment decisions tailored to patients' individual characteristics.

## 4 Discussion

Alzheimer's disease (AD) is a progressive neurodegenerative disorder frequently accompanied by multiple comorbidities that significantly impact patient outcomes and quality of life (1, 23). While substantial research has explored the effects of comorbidities on AD progression (9, 10, 23), less attention has been given to identifying the factors influencing the severity of comorbidities in these patients. Understanding these determinants is crucial for clinicians to develop personalized prevention and management strategies to mitigate the burden of comorbidities and enhance the quality of care.

With the rapid advancement of electronic medical records, large-scale datasets now allow for more robust and nuanced analyses of patient characteristics and outcomes. Machine learning models, with their powerful data processing and pattern recognition capabilities, have become increasingly valuable in personalized medicine. Utilizing data from the MIMIC-IV database, we aimed to identify factors influencing the severity of comorbidities in AD patients and constructed a visual predictive nomogram to assist in clinical decision-making.

Our study analyzed data from 507 AD patients with comorbidities. The age-adjusted Charlson Comorbidity Index (aCCI) was selected as the measure of comorbidity burden, dividing patients into aCCI-low and aCCI-high groups following established methods (12). Significant differences were observed in baseline characteristics between these groups, including age, Resprate, MCHC, Base Excess, Anion Gap, Glucose, RDW, Alkaline Phosphatase, Potassium (Whole Blood), Hematocrit, Urea Nitrogen, Phosphate, Creatinine, Hemoglobin, and MCH, with *P* < 0.05 for all. These findings suggested that the factors influencing aCCI were among these variables.

To address missing data in the MIMIC-IV database, we employed multiple imputation (MICE), a widely used approach for handling missing data under the assumption of missing at random (MAR). This method allowed us to generate plausible values for missing entries, reducing the potential bias associated with incomplete data. Importantly, we confirmed that the distributions of key variables, including aCCI, Age, and Glucose, remained consistent before and after imputation, supporting the validity of this approach. While multiple imputation may introduce some uncertainty, the use of LASSO regularization in feature selection minimized its impact on the results. Furthermore, the model's stable performance in the validation set (AUC: 0.757) demonstrated that the imputation process had a limited effect on the reliability of the findings.

We recognize that the threshold for dividing aCCI into low (≤ 5) and high (>5) groups could introduce ambiguity, particularly for patients with scores close to the cutoff. Sensitivity analysis indicated that slight variations in this boundary did not significantly affect the model's predictive performance or variable selection, suggesting that the grouping method was robust. Future studies could explore alternate thresholds or dynamic scoring systems to refine risk stratification further.

To further investigate, we incorporated these variables into both traditional multivariate logistic regression and four machine learning models: LASSO regression, Random Forest, XGBoost, and SVM. The dataset was randomly split into a 70% training set and a 30% validation set. LASSO regression demonstrated the best performance overall, filtering out 11 key feature factors while maintaining robust interpretability. Notably, the use of LASSO regularization effectively addressed multicollinearity among variables. Variance Inflation Factor (VIF) analysis of the selected features confirmed low multicollinearity (VIF < 5), which enhanced the stability and reliability of the model.

To address the potential for multicollinearity and variable interaction, exploratory analysis was conducted to assess interaction terms between key variables such as Age, Glucose, and Phosphate. The results did not identify significant interactions, suggesting that the predictive contributions of these variables were largely independent. Future studies could employ more sophisticated interaction analyses or advanced methods such as generalized additive models (GAMs) to explore these relationships further.

Age emerged as a critical determinant across all models, aligning with previous studies reporting a significant increase in comorbidity burden among AD patients aged over 80 years (24). In our study, the median age in the aCCI-low group was 81, compared to 85 in the aCCI-high group, reflecting the exacerbation of cardiovascular and metabolic risks with advanced age. Respiratory rate (Resprate) was another crucial feature, as an elevated Resprate may signal underlying cardiac insufficiency or heart failure, conditions commonly observed in AD patients with comorbid cardiovascular diseases (25–27).

The laboratory indicators identified by the LASSO model provided valuable clinical insights. Base Excess, a marker of acid-base balance, was negatively associated with aCCI, indicating higher risks of metabolic acidosis or alkalosis in patients with severe comorbidities (29–31). Glucose levels were positively associated with aCCI, highlighting the compounded risks of diabetes and cardiovascular complications in AD patients with elevated glucose (28, 32, 33). RDW, indicative of red blood cell deformability, was linked to increased risks of thrombosis and atherosclerosis, further exacerbating cardiovascular burdens (34–37). Phosphate and Alkaline Phosphatase levels were associated with kidney dysfunction and bone fragility, conditions that increase the likelihood of falls and fractures in elderly AD patients (38–40). Creatinine, a marker of renal function, reflected potential renal impairment, often comorbid with hypertension and diabetes in AD patients (46). Anemia-related indicators, such as Hematocrit and MCH, were negatively associated with aCCI, suggesting that anemic conditions contribute to cardiovascular strain and overall comorbidity risk (41–45).

To facilitate clinical application, we constructed a nomogram based on the selected variables to estimate the probability of high aCCI in AD patients. The nomogram's calibration and decision curve analyses demonstrated strong predictive accuracy and clinical utility. This tool allows clinicians to quickly identify high-risk patients and tailor individualized treatment plans.

This study also has important implications for clinical practice. By focusing on ICU patients with AD, it highlights the critical need for precise and efficient comorbidity burden assessment in this high-risk population. The aCCI serves as a practical and interpretable tool for quantifying disease complexity, enabling better individualization of management strategies and resource allocation in ICU settings. The identified features also provide insights into the pathophysiological mechanisms underlying comorbidity burden, offering potential avenues for intervention.

However, the findings may not generalize to non-ICU populations due to the nature of the MIMIC-IV dataset. External validation in independent cohorts, including non-ICU settings, is necessary to confirm the robustness of the model. Furthermore, although multiple imputation effectively addressed missing data in this study, future work should explore alternative methods, such as machine learning-based imputation, to enhance reliability in datasets with different missing data mechanisms. Longitudinal studies examining dynamic changes in aCCI over time could also provide valuable insights into the progression and management of comorbidities in AD patients.

Despite these limitations, this study demonstrates the potential of integrating machine learning approaches into clinical practice. By offering a reliable and interpretable method for evaluating comorbidity burden, it sets the stage for more personalized and effective management of AD patients in diverse healthcare settings.

## 5 Conclusions

The feature factors we identified were all closely related to the comorbidities in AD patients. The regression model constructed from these factors achieved an AUC of 0.762, demonstrating high calibration accuracy and significant net benefit. The nomogram based on this model effectively predicted the age-adjusted Charlson Comorbidity Index in patients and had the potential to be widely applied in clinical decision-making.

## Data Availability

The raw data supporting the conclusions of this article will be made available by the authors, without undue reservation. The code used in this study is available on GitHub at https://github.com/ALEX-DOCTOR123/github-pages. The code is openly accessible for other researchers to use and verify, under the applicable open-source license.
