# Supplementary material for: Study on influencing factors of age-adjusted Charlson comorbidity index in patients with Alzheimer's disease based on machine learning model
Source: Front Med (Lausanne). 2025 Jan 27;12:1497662. doi: 10.3389/fmed.2025.1497662 (PMC11807998; doi:10.3389/fmed.2025.1497662)
Supplement: Supplementary file 1 [file Table_1.docx]

| Variable | VIF |
| --- | --- |
| const | 601.2047251 |
| Creatinine | 1.315203594 |
| RDW | 1.29443356 |
| Phosphate | 1.27569364 |
| MCH | 1.18409719 |
| Hematocrit | 1.119221051 |
| Base Excess | 1.098570897 |
| Potassium | 1.092504135 |
| resprate | 1.055772059 |
| Alkaline Phosphatase | 1.046089788 |
| Glucose | 1.043238806 |
| age | 1.037279213 |

Supplementary Table 1. Variance Inflation Factor (VIF) Analysis of Selected Variables for Multicollinearity Assessment.
