# Supplementary material for: Study on influencing factors of age-adjusted Charlson comorbidity index in patients with Alzheimer's disease based on machine learning model
Source: Front Med (Lausanne). 2025 Jan 27;12:1497662. doi: 10.3389/fmed.2025.1497662 (PMC11807998; doi:10.3389/fmed.2025.1497662)
Supplement: Supplementary file 2 [file Table_2.docx]

**Table S2: Baseline characteristics table of patients**

|  | **ALL** | **aCCI-low** | **aCCI-high** | ***P* overall** | **Adjusted_P_value** |
| --- | --- | --- | --- | --- | --- |
|  | ***n=507*** | ***n=109*** | ***n=398*** |  |  |
| Age | 85.00 [79.00;89.50] | 81.00 [74.00;89.00] | 85.00 [80.00;90.00] | 0.009 | 0.054 |
| Gender: |  |  |  | 0.914 | 0.968 |
| F | 293 (57.79%) | 62 (56.88%) | 231 (58.04%) |  |  |
| M | 214 (42.21%) | 47 (43.12%) | 167 (41.96%) |  |  |
| Heartrate | 84.00 [71.50;100.00] | 82.00 [71.00;97.00] | 86.00 [72.00;100.00] | 0.145 | 0.337 |
| Resprate | 18.00 [16.00;21.00] | 18.00 [16.00;19.00] | 18.00 [16.00;21.00] | 0.001 | 0.009 |
| SBP | 118.00 [105.00;136.50] | 114.00 [105.00;137.00] | 118.00 [105.00;136.00] | 0.922 | 0.968 |
| DBP | 67.00 [57.00;76.00] | 68.00 [58.00;77.00] | 67.00 [56.25;75.00] | 0.279 | 0.502 |
| GCS | 12.00 [8.00;14.00] | 11.00 [7.00;13.00] | 12.00 [8.00;14.00] | 0.121 | 0.327 |
| SOFA | 5.00 [3.00;7.00] | 5.00 [3.00;7.00] | 5.00 [3.00;7.00] | 0.635 | 0.809 |
| SIRS | 3.00 [2.00;3.00] | 3.00 [2.00;3.00] | 3.00 [2.00;3.00] | 0.679 | 0.833 |
| LODS | 5.00 [3.00;7.00] | 5.00 [3.00;6.00] | 5.00 [4.00;8.00] | 0.066 | 0.198 |
| aCCI | 7.00 [6.00;8.00] | 5.00 [5.00;5.00] | 7.00 [6.00;9.00] | <0.001 | <0.001 |
| Alanine Aminotransferase(ALT) | 20.00 [14.00;31.50] | 19.00 [14.00;30.00] | 20.00 [14.00;32.00] | 0.850 | 0.956 |
| Platelet Count | 217.00 [174.50;276.00] | 211.00 [166.00;276.00] | 217.50 [177.00;276.00] | 0.636 | 0.809 |
| MCHC | 32.90 [31.80;33.70] | 33.00 [32.00;34.30] | 32.80 [31.70;33.60] | 0.032 | 0.108 |
| Base Excess | 0.00 [-2.00;1.00] | 0.00 [-1.00;2.00] | 0.00 [-3.00;1.00] | 0.022 | 0.079 |
| PH | 6.50 [5.50;7.28] | 6.50 [6.00;7.35] | 6.50 [5.50;7.24] | 0.137 | 0.336 |
| Anion Gap | 15.00 [13.00;17.00] | 14.00 [13.00;16.00] | 15.00 [13.00;18.00] | 0.016 | 0.072 |
| Creatine Kinase(CK) | 97.00 [51.00;194.00] | 100.00 [51.00;235.00] | 95.50 [51.00;182.00] | 0.644 | 0.809 |
| MCV | 92.00 [88.00;96.00] | 92.00 [89.00;96.00] | 92.00 [87.00;96.00] | 0.111 | 0.315 |
| Glucose | 125.00 [102.00;164.50] | 113.00 [93.00;134.00] | 129.00 [103.25;181.00] | <0.001 | <0.001 |
| RDW | 14.10 [13.30;15.10] | 13.90 [13.20;14.60] | 14.20 [13.40;15.17] | 0.003 | 0.02 |
| PT | 12.70 [11.80;14.20] | 12.80 [11.90;13.70] | 12.70 [11.80;14.30] | 0.978 | 0.978 |
| Calcium(Total) | 8.90 [8.30;9.40] | 8.80 [8.20;9.10] | 8.90 [8.40;9.40] | 0.051 | 0.162 |
| Red Blood Cells | 4.08 [3.63;4.51] | 4.13 [3.73;4.51] | 4.05 [3.62;4.51] | 0.214 | 0.413 |
| Calculated (Total CO2) | 25.00 [22.00;28.00] | 26.00 [23.00;28.00] | 25.00 [22.00;28.00] | 0.127 | 0.327 |
| Alkaline Phosphatase | 82.00 [64.00;111.00] | 75.00 [60.00;96.00] | 84.00 [65.00;111.00] | 0.011 | 0.059 |
| Potassium(Whole Blood) | 4.20 [3.70;4.70] | 4.10 [3.60;4.50] | 4.20 [3.80;4.80] | 0.019 | 0.073 |
| White Blood Cells | 9.00 [6.60;12.20] | 9.10 [7.00;12.40] | 8.80 [6.40;12.17] | 0.466 | 0.664 |
| Monocytes | 5.30 [4.00;7.00] | 5.30 [4.40;7.00] | 5.30 [3.90;7.00] | 0.450 | 0.664 |
| Lactate Dehydrogenase(LD) | 231.00 [192.00;299.00] | 240.00 [199.00;302.00] | 227.00 [191.00;299.00] | 0.153 | 0.337 |
| Asparate Aminotransferase(AST) | 26.00 [20.00;38.00] | 25.00 [22.00;42.00] | 26.00 [19.00;36.00] | 0.191 | 0.386 |
| Lactate | 1.80 [1.30;2.60] | 1.80 [1.30;2.40] | 1.80 [1.30;2.60] | 0.763 | 0.877 |
| Magnesium | 2.00 [1.80;2.20] | 2.00 [1.80;2.20] | 2.00 [1.80;2.20] | 0.423 | 0.664 |
| Specific Gravity | 1.02 [1.01;1.02] | 1.02 [1.01;1.02] | 1.02 [1.01;1.02] | 0.363 | 0.613 |
| Sodium | 140.00 [137.00;143.00] | 140.00 [137.00;142.00] | 140.00 [137.00;143.00] | 0.379 | 0.62 |
| Basophils | 0.30 [0.20;0.50] | 0.30 [0.20;0.50] | 0.30 [0.20;0.50] | 0.633 | 0.809 |
| pO2 | 82.00 [48.00;194.50] | 113.00 [51.00;194.00] | 80.50 [48.00;194.00] | 0.193 | 0.386 |
| Potassium | 4.20 [3.90;4.70] | 4.20 [3.80;4.60] | 4.30 [3.90;4.70] | 0.250 | 0.466 |
| Hematocrit | 37.20 [33.50;40.60] | 38.90 [34.60;41.10] | 36.95 [33.32;40.40] | 0.018 | 0.073 |
| Urea Nitrogen | 23.00 [17.00;31.50] | 19.00 [16.00;25.00] | 23.00 [17.00;34.00] | <0.001 | <0.001 |
| Bilirubin(Total) | 0.50 [0.30;0.80] | 0.50 [0.40;0.80] | 0.50 [0.30;0.70] | 0.156 | 0.337 |
| Hemoglobin | 12.20 [11.00;13.40] | 12.70 [11.60;13.70] | 12.00 [10.70;13.30] | 0.003 | 0.02 |
| Phosphate | 3.40 [2.80;3.90] | 3.10 [2.60;3.70] | 3.40 [2.90;4.00] | <0.001 | <0.001 |
| Lymphocytes | 14.05 [8.30;22.00] | 14.05 [8.00;21.50] | 14.05 [8.50;22.08] | 0.761 | 0.877 |
| Albumin | 3.60 [3.10;4.10] | 3.70 [3.20;4.10] | 3.60 [3.10;4.10] | 0.467 | 0.664 |
| Chloride | 103.00 [100.00;106.00] | 103.00 [100.00;106.00] | 103.00 [100.00;106.00] | 0.739 | 0.386 |
| Neutrophils | 77.30 [67.25;84.90] | 77.70 [68.20;86.00] | 76.95 [67.23;84.20] | 0.446 | 0.877 |
| INR/PT | 1.10 [1.00;1.30] | 1.10 [1.10;1.20] | 1.10 [1.00;1.30] | 0.932 | 0.968 |
| Creatinine | 1.00 [0.80;1.40] | 0.90 [0.80;1.10] | 1.10 [0.80;1.40] | <0.001 | <0.001 |
| PTT | 28.30 [25.30;31.35] | 28.30 [24.50;30.90] | 28.30 [25.70;31.40] | 0.304 | 0.53 |
| Bicarbonate | 25.00 [22.00;27.50] | 25.00 [23.00;27.00] | 25.00 [22.00;28.00] | 0.881 | 0.968 |
| Eosinophils | 0.80 [0.20;2.10] | 0.70 [0.20;2.00] | 0.90 [0.20;2.10] | 0.623 | 0.809 |
| MCH | 30.20 [28.80;31.50] | 30.60 [29.50;31.80] | 30.10 [28.60;31.50] | 0.015 | 0.072 |
| pCO2 | 40.00 [35.00;47.00] | 39.00 [35.00;47.00] | 40.00 [35.00;47.00] | 0.954 | 0.972 |
