# Supplementary figures and images for: Study on influencing factors of age-adjusted Charlson comorbidity index in patients with Alzheimer's disease based on machine learning model
Source: Front Med (Lausanne). 2025 Jan 27;12:1497662. doi: 10.3389/fmed.2025.1497662 (PMC11807998; doi:10.3389/fmed.2025.1497662)

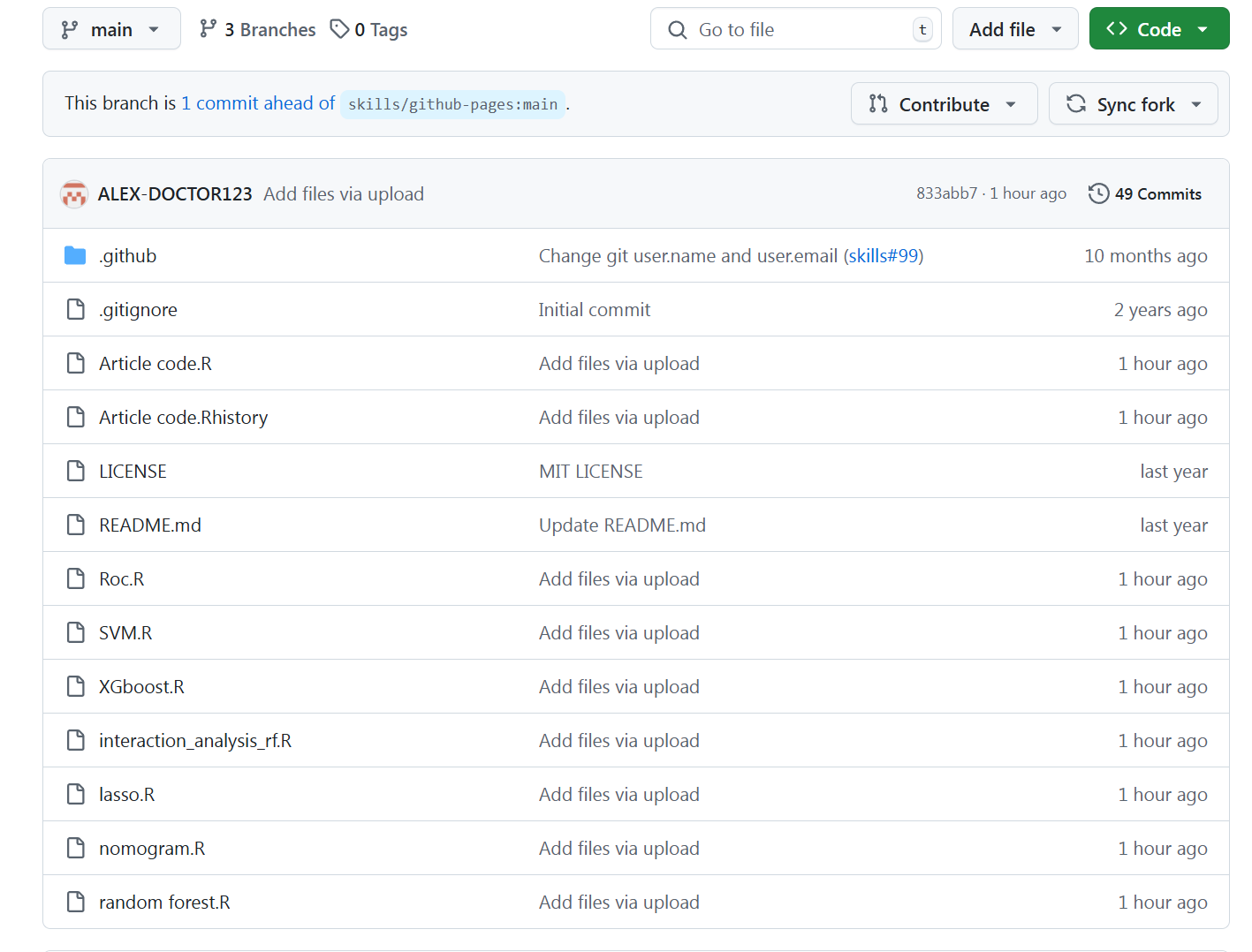

Supplement: Supplementary file 3 [file Image_1.png]

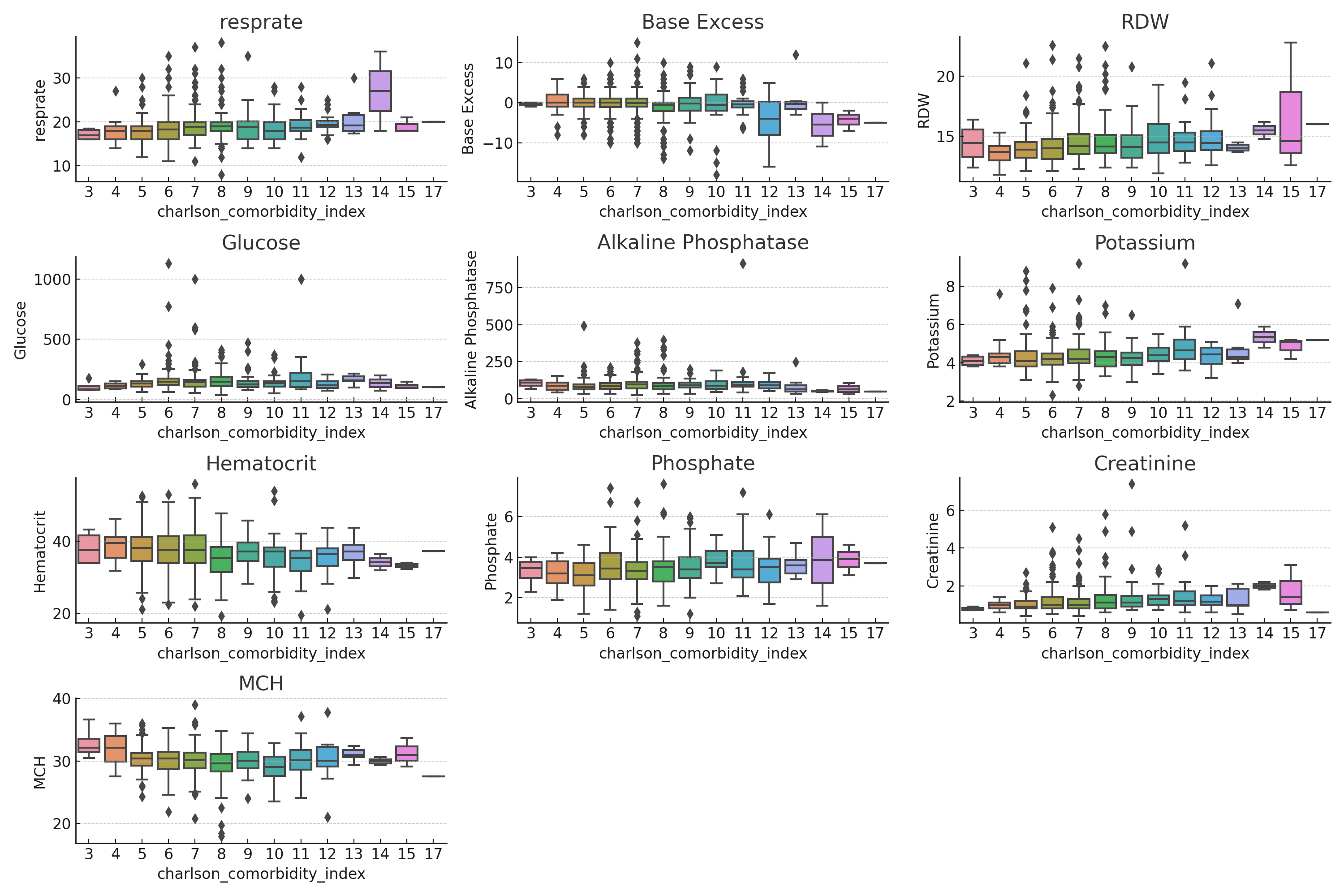

Supplement: Supplementary file 4 [file Image_2.png]

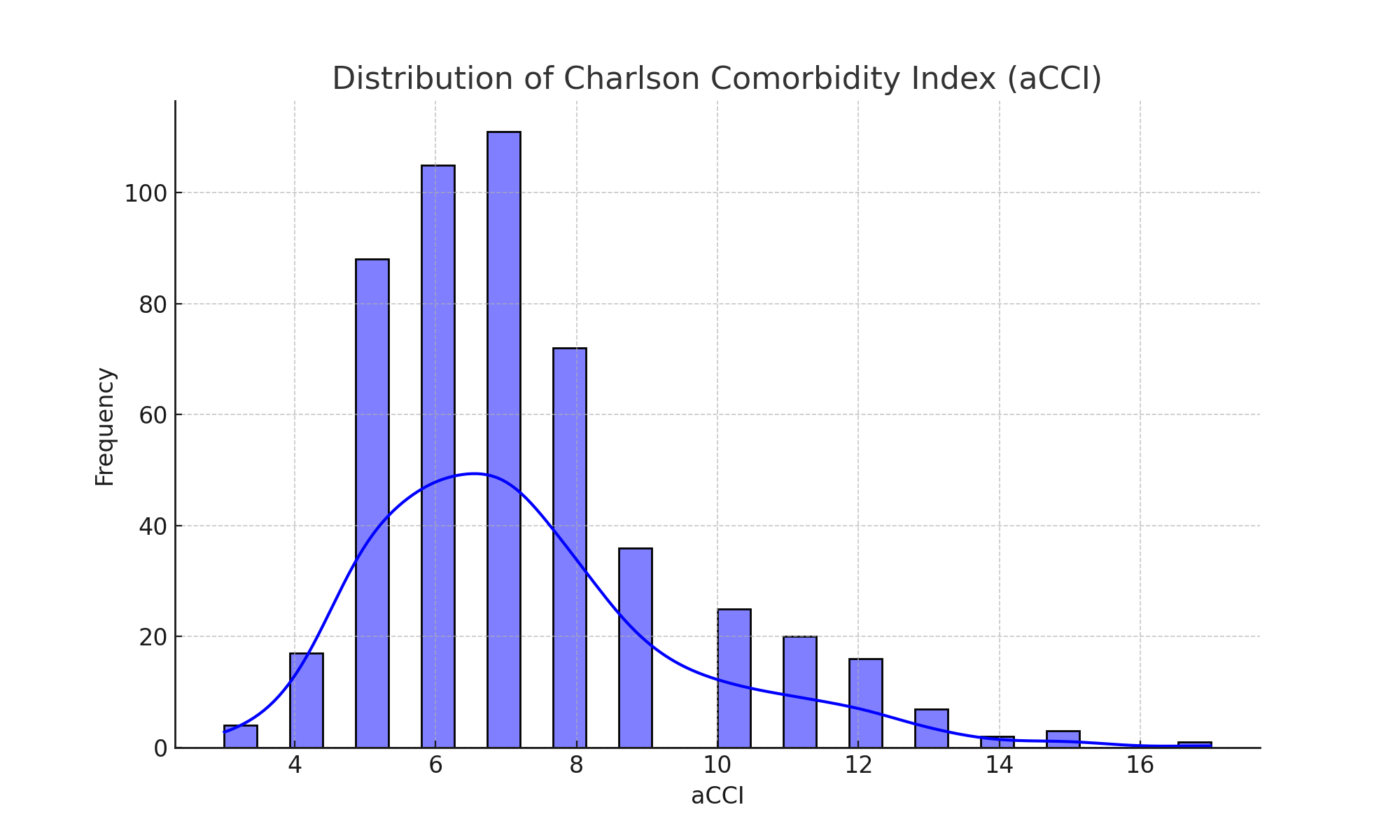

Supplement: Supplementary file 5 [file Image_3.png]
